# Hearing impairment risk and interaction of folate metabolism related gene polymorphisms in an aging study

**DOI:** 10.1186/1471-2350-12-35

**Published:** 2011-03-07

**Authors:** Yasue Uchida, Saiko Sugiura, Fujiko Ando, Tsutomu Nakashima, Hiroshi Shimokata

**Affiliations:** 1 Department of Otorhinolaryngology, National Center for Geriatrics and Gerontology, 35 Gengo, Morioka, Obu City, Aichi Prefecture, 474-8511, Japan; 2 Department of Health and Medical Sciences, Aichi Shukutoku University, 9 Katahira, Nagakute, Nagakute-cho, Aichi-gun, Aich Prefecture, 480-1197, Japan; 3 Department for Development of Preventive Medicine, Center for Development of Advanced Medicine for Dementia, National Center for Geriatrics and Gerontology, 35 Gengo, Morioka, Obu city, Aichi prefecture, 474-8511, Japan; 4 Department of Otorhinolaryngology, Nagoya University School of Medicine, 65 Tsuruma-Cho, Showa Ward, Nagoya City, Aichi Prefecture, 466-8550, Japan

## Abstract

**Background:**

Recent investigations demonstrated many genetic contributions to the development of human age-related hearing impairment (ARHI), however, reports of factors associated with a reduction in the ARHI risk are rare. Folate metabolism is essential for cellular functioning. Despite the extensive investigations regarding the roles of folate metabolism related gene polymorphisms in the pathophysiology of complex diseases, such as cancer, cardio-cerebrovascular disease, and atherosclerosis, little is known about the association with ARHI. The aim of this study is to investigate the effects of the methionine synthase (MTR) A2756G and methylenetetrahydrofolate reductase (MTHFR) C677T gene polymorphisms on the risk of hearing impairment in middle-aged and elderly Japanese.

**Methods:**

Data were collected from community-dwelling Japanese adults aged 40-84 years who participated in the Longitudinal Study of Aging biennially between 1997 and 2008. We analyzed cumulative data (5,167 samples in accumulated total) using generalized estimating equations.

**Results:**

The MTHFR 677T allele was significantly associated with a reduced risk of hearing impairment only when the subjects were wild-type homozygotes for MTR A2756G. The per-T allele odds ratio of MTHFR for the risk of developing hearing impairment was 0.7609 (95% CI: 0.6178-0.9372) in the MTR AA genotype. In addition, a subgroup analysis demonstrated that the favorable effect of the MTHFR 677T allele on the risk of developing hearing impairment was independent of folate and homocysteine level, whereas plasma total homocysteine level was independently associated with an increased risk of developing hearing impairment. The interactive effect of gene polymorphisms associated with folate metabolism may modify the risk of developing hearing impairment after middle age. These results contribute to the elucidation of the causes of ARHI.

**Conclusions:**

The present study has found that the MTHFR 677T allele has a favorable effect on a risk of hearing impairment in the middle-aged and elderly population, only when the individuals were wild-type homozygotes for MTR A2756G.

## Background

Presbycusis is one of the most common sensory impairments affecting the elderly and is a multifactorial process that involves a multitude of intrinsic and extrinsic factors. Recent investigations demonstrated many genetic contributions to human age-related hearing impairment (ARHI) [1-7].

Folate metabolism is essential for cellular functioning because it provides one-carbon donors for the *de novo *synthesis of purines and pyrimidines, which are necessary for RNA and DNA synthesis, and provides methyl groups, which are necessary for the remethylation of homocysteine and for methylation reactions such as DNA methylation [8,9]. The enzyme methylenetetrahydrofolate reductase (MTHFR) irreversibly catalyzes the conversion of 5,10-methylenetetrahydrofolate (5,10-methyleneTHF) to 5-methyltetrahydrofolate (5-methylTHF), a methyl donor. Methionine synthase (MTR) catalyzes the remethylation of homocysteine to methionine. Folate in the form of 5,10-methyleneTHF donates a methyl group to uracil, converting it to thymidine, which is used for DNA synthesis and repair [10]. (See Additional file 1, Figure S1.)

Folates cannot be synthesized *de novo *by mammals. Polymorphisms in genes encoding critical enzymes in folate metabolism play important and interrelated roles in the pathophysiology of cancer [9-13], cardio-cerebrovascular disease, atherosclerosis [14], pregnancy loss, renal failure [15], diabetic retinopathy [16], and the progression of Alzheimer's disease [17].

The MTHFR C677T polymorphism (rs1801133), which results in an alanine-to-valine substitution, is one of the most extensively investigated functional polymorphisms of genes encoding one-carbon metabolism enzymes and occurs frequently in Caucasian and Asian populations. This substitution results in a 30% decrease in MTHFR activity in heterozygotes and a 60% decrease in MTHFR activity in homozygotes [18].

Although reports are not uniformly consistent, this polymorphism has been shown to modify the risk of developing several cancers in a site-specific manner [13,19,20]. It decreases the risk of colorectal cancer, hepatocellular carcinoma, cervical cancer, and certain leukemias and lymphomas. In contrast, it increases the risk of cancer of the breast, endometrium, esophagus, stomach, pancreas, and bladder [13]. Epidemiological evidence indicates that this polymorphism has a protective effect against colorectal cancer in individuals with an adequate or high status of folate and nutrients involved in one-carbon metabolism [21-23].

The most common MTR gene polymorphism is MTR A2756G (rs1805087), which results in a substitution of aspartic acid for glycine and decreases methionine synthase activity [24]. This polymorphism increases the cellular homocysteine level [25], resulting in DNA hypomethylation [26].

The MTHFR and MTR polymorphisms have attracted interest as causes of sudden sensorineural hearing loss (SSNHL) [27-29]. We reported results from a nested case-control study using data from the National Institute for Longevity Sciences - Longitudinal Study of Aging (NILS-LSA) that showed that the T allele of MTHFR C677T is associated with susceptibility to SSNHL. However, little has been reported on the association between folate pathway gene polymorphisms and ARHI. Durga et al. investigated the effects of folate status and the MTHFR C677T polymorphism on hearing in middle-aged and elderly subjects [30].

In this study, we investigated whether the MTHFR C677T (rs1801133) and MTR A2756G (rs1805087) polymorphisms are associated with hearing impairment in middle-aged and elderly people in a Japanese community.

## Methods

### Subjects

The subjects used in this study were derived from the NILS-LSA, an ongoing population-based biennial survey of a cohort of approximately 2,200 persons. The NILS-LSA is a comprehensive and interdisciplinary study to observe age-related changes and consists of various gerontological and geriatric measurements, such as medical examinations, blood chemical analysis, body composition, anthropometry, physical function, nutritional analysis, psychological tests, and visual and auditory function. The subjects of the NILS-LSA were randomly selected from resident registrations, stratified by both age and sex. The numbers of men and women recruited were similar, and age at baseline was 40 years to 79 years, with similar numbers of participants in each decade of age (40s, 50s, 60s, and 70s). Details of the NILS-LSA are described elsewhere [5]. The study protocol was approved by the Ethical Committee of the National Center for Geriatrics and Gerontology and written informed consent was obtained from all participants. Those who did not consent to have blood samples taken and those who did not complete the hearing test were excluded. As the MTHFR C677T and MTR A2756G polymorphisms can be associated with SSNHL, participants who reported a history of ear disease were also excluded. Gene polymorphisms were examined using blood samples taken at the initial examination. The baseline subjects of this study were 1,426 adults (722 men and 704 women) aged 40-79 years who completed the first-wave examinations of NILS-LSA between November 1997 and April 2000. Of these, 1,103 (77.3%) took part in the second-wave examination, 994 (69.7%) took part in the third-wave examination, 855 (60.0%) participated in the fourth-wave examination, and 789 (55.3%) participated in the fifth-wave examination. The mean number of repeat visits was 3.6. The total number of visits, including repeat visits, was 5,167; the participants from whom the data were derived were 40-84 years of age and took part in the NILS-LSA between November 1997 (the first wave) and July 2008 (the fifth wave).

The genotype frequencies, in participants of the NILS-LSA at baseline examination both before and after employing exclusion criteria of the present analyses, were not significantly different from those expected based on the Hardy-Weinberg equilibrium in the MTHFR C677T and MTR A2756G polymorphisms (chi-square test, P > .05).

#### Ethical approval

Ethical Committee of the National Center for Geriatrics and Gerontology.

Identification number: #14, #52, #74.

### Genetic analyses

Genomic DNA was extracted from peripheral blood lymphocytes using a standard procedure. The genotype (A or G) at position 2756 of the MTR gene and the genotype (C or T) at position 677 of the MTHFR gene were determined using an allele-specific-primer polymerase chain reaction (ASP-PCR) method (Toyobo Gene Analysis, Tsuruga, Japan).

#### • **MTHFR genotype analysis**

The single nucleotide polymorphism region of the gene was amplified by PCR using two allele-specific-primers (ASPs): (C-specific primer, 5'-GAAGGTGTCTGCGGGAXCC-3'; T-specific primer, 5'-GAGAAGGTGTCTGCGGGAXTC-3') and a biotin-labeled common antisense primer (5'-biotin-GAATGTGTCAGCCTCAAAGAAA-3'). Amplified allele-specific DNA products were used for colorimetric genotyping. For MTHFR genotyping, two types of wells conjugated with the allele-specific C-type probe (5'-TCTGCGGGAXCCGATTTCAT-3') or T-type probe (5'-TCTGCGGGAXTCGATTTCAT-3') were prepared. The amplified DNA product was denatured with NaOH and added to each well. Then, it was hybridized at 37°C for 30 minutes with hybridization buffer containing formamide. After the wells were thoroughly washed, alkaline phosphatase-conjugated streptavidin was added to each well, and the plate was incubated at 37°C for 15 minutes. After the wells were washed, 0.8 mmol/L WST-1 [2-(4-iodophenyl)-3-(4-nitrophenyl)-5-(2,4-di-sulfophenyl)-2H-tetrazolium, monosodium salt] and 0.4 mmol/L BCIP (5-bromo-4-chloro-3-indolyl phosphate p-toluidine salt), a substrate for alkaline phosphatase, were added and colorimetry was performed. The genotypes were identified according to the absorbance signal ratio between C type-specific and T type-specific wells. Further details of MTHFR genotyping in the NILS-LSA are supplied elsewhere [31].

#### • **MTR genotype analysis**

The polymorphic region of the gene was amplified using PCR and two ASPs labeled at the 5" end, either with fluorescein isothiocyanate (A-specific primer, 5'-GGAAGAATATGAAGATATTAGACAGXAC-3') or with Texas Red (G-specific primer, 5'-GAAGAATATGAAGATATTAGACAGXGC-3') and a biotin-labeled common antisense primer (5'-biotin-CTACCACTTACCTTGAGAGACTCAT-3'). The reaction mixture (25 μL) contained 20 ng of DNA, 5 pmol of each primer, 0.2 mmol/L of each deoxynucleoside triphosphate, 5.0 mmol/L MgCl_2_, and 1 U of rTaq DNA polymerase (Toyobo, Osaka, Japan) in the appropriate DNA polymerase buffer. The amplification protocol consisted of an initial denaturation at 95°C for 5 minutes, 35 cycles of denaturation at 95°C for 30 seconds, annealing at 65°C for 30 seconds, extension at 72°C for 30 seconds, and a final extension at 72°C for 2 minutes. Amplified DNA was incubated in a solution containing streptavidin-conjugated magnetic beads in the wells of a 96-well plate at room temperature. The plate was placed on a magnetic stand, and the supernatants were then collected from each well, transferred to the wells of a 96-well plate containing 0.01 mol/L NaOH, and fluorescence was then measured using a microplate reader (Spectra Fluor; Tecan Co., Tokyo, Japan) at excitation and emission wavelengths of 485 nm and 538 nm, respectively, for fluorescein isothiocyanate and 584 nm and 612 nm, respectively, for Texas Red.

### Measures

Air-conduction pure-tone thresholds at octave intervals from 0.5 kHz to 8 kHz were measured in a sound-proof booth by trained examiners using a standardized protocol and a diagnostic audiometer (AA-73A and AA-78; Rion, Tokyo, Japan). For analyses of supraliminal levels, thresholds in excess of the predetermined output level according to the JIS (Japanese Industrial Standards T 1201) calibration plus an additional 5 dB were treated as levels. The average hearing threshold level (AHT) of the better ear at frequencies of 0.5, 1, 2, and 4 kHz was used as an index of hearing status among genotypes. Hearing impairment was defined as an AHT greater than 25 dB according to World Health Organization grades [32].

Venous blood samples were collected with the consent of the participants for genetic and other blood analyses. Blood samples were collected early in the morning after at least 12 hours of fasting. Plasma total homocysteine concentration was determined using high-performance liquid chromatography and fluorimetric detection [33]. The serum concentration of folate was determined using a chemiluminescent enzymatic reaction using Access (Beckman Coulter Inc., Fullerton, CA).

Most of the moderator variables for analyses (history of occupational noise exposure, lifetime smoking, heart disease, hypertension, and diabetes) were self-reported. Histories of occupational noise exposure and lifetime smoking were categorized as never vs. ever. Individual medical histories were obtained using questionnaires. Each of the cited moderators was treated as a binary variable (presence vs. absence). Body mass index (BMI) was calculated as weight (kg) divided by height (m^2^).

### Statistical analysis

Statistical analyses were conducted using Statistical Analysis System version 9.1.3 (SAS Institute, Cary, NC). Univariate analyses of categorical variables according to hearing impairment status were performed using the chi-square test and baseline data. The chi-square test was also conducted to assess the independency of genotype distribution of the two polymorphisms in the baseline data. Cumulative data were analyzed using generalized estimating equations (GEEs), which take into account the dependency of repeated observations within subjects. GEE models were fitted using the GENMOD procedure of SAS. The GENMOD procedure fits generalized linear models. The correlation structure was specified as autoregressive.

Genotypes were coded as follows: wild-type homozygotes, AA for MTR and CC for MTHFR, heterozygotes, AG for MTR and CT for MTHFR, and mutant homozygotes, GG for MTR and TT for MTHFR. In an exploratory analysis to assess the effect of MTR A2756G and MTHFR C677T on hearing impairment, MTR, MTHFR, and the MTHFR by MTR interaction were set as independent variables using the GENMOD procedure. Then, based on the results of the exploratory analysis, odds ratios (ORs) for the additive genetic models of MTHFR polymorphisms according to MTR genotypes with the risk of hearing impairment were analyzed with adjustment for age, sex, smoking status, BMI, and histories of occupational noise exposure, heart disease, hypertension, and diabetes. The additive genetic model, which is the prevailing analytical model in genetic epidemiology, assumes that there is a linear gradient in risk with increasing numbers of mutated alleles. The per-allele OR for the risk of developing hearing impairment was determined using the additive genetic model.

Data from only 1,146 of the 1,426 baseline participants could be used for a subgroup analysis in which blood folate and homocysteine levels were included as variables in the statistical model because blood folate and homocysteine levels were measured during first-wave examinations. Comparison of serum folate and plasma total homocysteine levels according to MTHFR genotypes by MTR genotypes was conducted using a general linear model. A multiple logistic regression was then performed to obtain ORs for hearing impairment in subjects with the MTHFR C677T polymorphism by MTR genotypes as a subgroup analysis. In this analysis, age, sex, smoking status, BMI, and histories of occupational noise exposure, heart disease, hypertension, and diabetes were denoted as possible influential variables. ORs per ng/mL increase in serum concentration of folate and per nmol/mL increase in plasma total homocysteine concentration were also estimated.

All values are expressed as the mean ± standard deviation unless otherwise specified. A null-hypothesis probability of < 0.05 was regarded as statistically significant.

## Results

The general characteristics and genotype frequencies of the baseline subjects are presented in Table 1 according to hearing impairment status. As stated before, participants who reported a history of ear disease were excluded since the MTHFR C677T and MTR A2756G polymorphisms are associated with SSNHL. No significant differences in hearing impairment frequency were observed between the MTR and MTHFR genotypes according to univariate analysis. As expected, the frequency of hearing impairment was greater in males than in females. The prevalence of hearing impairment was greater in subjects who had histories of occupational noise exposure, heart disease, hypertension, and diabetes, and smoking and in older subjects. The distributions of MTR A2756G and MTHFR C677T at baseline were not significantly associated with each other (chi-square test, *p *> 0.05).

**Table 1:** General characteristics and genotype frequencies at baseline according to hearing impairment status.

Variable	Total N (%)	Hearing impairment		No hearing impairment		
		N	%	N	%	*p*
	1426	275	19.3	1151	80.7	
MTR A2756G						
AA (wild-type homozygotes)	939 (65.8)	174	18.5	765	81.5	0.2459
AG (heterozygotes)	436 (30.6)	94	21.6	342	78.4	
GG (mutant homozygotes)	51 (3.6)	7	13.7	44	86.3	

A allele	81.1%	442	19.1	1872	80.9	-
G allele	18.9%	108	20.1	430	79.9	

MTHFR C677T						
CC (wild-type homozygotes)	529 (37.1)	115	21.7	414	78.3	0.1954
CT (heterozygotes)	681 (47.8)	122	17.9	559	82.1	
TT (mutant homozygotes)	216 (15.1)	38	17.6	178	82.4	

C allele	61.0%	352	20.2	1387	79.8	-
T allele	39.0%	198	17.8	915	82.2	

Gender						
Male		173	24.0	549	76.0	<.0001
Female		102	14.5	602	85.5	

Age						
<60		19	2.8	660	97.2	<.0001
≥60		256	34.3	491	65.7	

Occupational noise exposure						
Yes		111	37.4	186	62.6	<.0001
No		164	14.5	965	85.5	

History of hypertension						
Yes		98	26.8	268	73.2	<.0001
No		177	16.7	883	83.3	

History of heart disease						
Yes		42	27.3	112	72.7	0.0078
No		233	18.3	1039	81.7	

History of diabetes						
Yes		41	35.3	75	64.7	<.0001
No		234	17.9	1076	82.1	

Smoking						
Current and ex-smoker		144	23.0	481	77.0	0.0015
Nonsmoker (Never smoked)		131	16.4	670	83.6	

Obesity						
BMI ≥ 25		55	17.4	261	82.6	0.3371
BMI < 25		220	19.8	890	80.2	

N = 1426 (Age, mean ± SD: 59.8 ± 10.9)

The genotype frequencies of the two polymorphisms in the cumulative data are presented according to hearing impairment status in Table 2. Hearing impairment frequencies were 15.8% for the MTR AA genotype, 30.1% for the MTR AG genotype, and 37.2% for the MTR GG genotype. An exploratory analysis of the effects of MTR, MTHFR, and the MTHFR by MTR interaction on hearing impairment with no adjustments conducted using the GENMOD procedure showed that the main effects of MTR and MTHFR and the interaction effect were significant (*p *= 0.0318, 0.0022, and 0.0076, respectively). After adjustment for moderators (age, sex, smoking status, BMI, history of occupational noise exposure, heart disease, hypertension, and diabetes), the main effect of MTHFR was still significant (*p *= 0.0230), but the main effect of MTR was not significant (*p *= 0.2736). The interactive effect was almost significant (*p *= 0.0856). The per-allele OR for the risk of developing hearing impairment according to the additive genetic model of MTHFR polymorphisms by MTR genotypes with adjustment for moderators is shown in Table 3 with 95% confidence intervals (CIs). The per-T allele OR of MTHFR for the risk of developing hearing impairment was 0.7609 (95% CI: 0.6178-0.9372) in the MTR AA genotype. The risk of hearing impairment decreased significantly with an increase in the frequency of the T allele of MTHFR independently of moderators, but only in the MTR AA genotype. Per-T allele ORs were not statistically significant for other MTR genotypes.

**Table 2:** Genotype frequencies of two polymorphisms in cumulative data according to hearing impairment status.

MTR	MTHFR	Hearing impairment		No hearing impairment	
A2756G	C677T	N	%	N	%
					
	CC	317	25.7	915	74.3
AA	CT	354	21.4	1297	78.6
	TT	77	15.8	410	84.2

	CC	137	21.5	501	78.5
AG	CT	184	24.6	564	75.4
	TT	69	30.1	160	69.9

	CC	16	25.4	47	74.6
GG	CT	10	13.2	66	86.8
	TT	16	37.2	27	62.8

Total N		1180	22.8	3987	77.2

N = 5167

**Table 3:** Odds ratios and 95% confidence intervals for additive genetic model of MTHFR polymorphism by MTR genotypes with risk of hearing impairment analyzed by generalized estimating equations in cumulative data.

MTR		MTHFR			
		CC (T = 0)	CT (T = 1)	TT (T = 2)	*p*
	N	1933	2475	759	
**AA (wild-type homozygotes)**	**3370**	**1**	**0.7609 (0.6178-0.9372)/T-allele**		**0.0102**
AG (heterozygotes)	1615	1	1.1040 (0.7963-1.5305)/T-allele		0.5528
GG (mutant homozygotes)	182	1	1.3231 (0.6332-2.7649)/T-allele		0.4565

N = 5167

Parenthetical reference shows 95% confidence interval.

[Moderator variable]

age, sex, smoking status, BMI

histories of occupational noise exposure, heart disease, hypertension, and diabetes

In a subgroup analysis, the mean level of serum folate in 1,146 subjects was 5.6 ± 2.6 ng/mL, and that of plasma total homocysteine was 11.0 ± 5.1 nmol/mL. Levels of serum folate and plasma total homocysteine according to MTR and MTHFR genotypes are shown in Table 4. Serum folate levels were significantly different among the MTHFR genotypes in the heterozygous and homozygous MTR 2756A genotypes, and levels of plasma total homocysteine differed significantly among the MTHFR genotypes in all MTR genotypes. The results of multiple logistic regression are presented in Table 5. In the MTR 2756A homozygous genotype, the MTHFR 677T allele showed an effect opposite to a high homocysteine level. The MTHFR 677T allele was significantly associated with a reduction in the risk of developing hearing impairment when associated with the MTR 2756AA genotype; this association was independent of folate or homocysteine level. Homocysteine level was associated with an increase in the risk of hearing impairment, but folate level was not. No significant association with a risk of hearing impairment was observed under conditions of the MTR 2756AG genotype. Data for the MTR 2756GG genotype were discarded because the small number of cases rendered the analysis of dubious validity.

**Table 4:** Comparison of serum folate and plasma total homocysteine levels according to MTR and MTHFR genotypes.

		**MTHFR**			
	**MTR**	**CC**	**CT**	**TT**	***p***
	
	AA	6.0 ± 2.6	5.6 ± 2.6	4.8 ± 2.1	<.0001
folate	AG	6.0 ± 3.2	5.7 ± 2.1	4.8 ± 2.0	0.0159
(ng/mL)	GG	6.5 ± 3.2	5.3 ± 2.2	4.1 ± 1.8	0.0970
	
	AA	10.5 ± 3.3	10.6 ± 3.9	13.8 ± 10.6	<.0001
homocysteine	AG	10.0 ± 2.5	10.9 ± 3.8	13.2 ± 8.8	0.0001
(nmol/mL)	GG	8.9 ± 1.7	10.1 ± 2.2	15.0 ± 6.8	0.0006

N = 1146

**Table 5:** Odds ratios and 95% confidence intervals for additive genetic model of MTHFR polymorphism by MTR genotypes, folate and homocysteine with risk of hearing impairment analyzed by a multiple logistic regression in a subgroup analysis.

	No.		ORs (95% confidence interval)		
MTR	Hearing impairment	No hearing impairment	MTHFR/T-allele	folate	homocysteine
AA	126	626	0.602 *	0.946	1.066 *
			(0.421-0.861)	(0.861-1.039)	(1.021-1.113)
					
AG	71	281	1.379	0.941	0.953
			(0.830-2.292)	(0.816-1.084)	(0.893-1.017)
					
GG	7	35	Data not shown (the validity of the model fit is questionable due to small number).		

N = 1146

Asterisk shows statistically significance (p < 0.05). Parenthetical reference shows 95% confidence interval.

[Moderator variable]

age, sex, smoking status, BMI

histories of occupational noise exposure, heart disease, hypertension, and diabetes

## Discussion

The main findings of our study were that the risk of developing hearing impairment in middle-aged and elderly subjects is influenced by an interaction between the MTR A2756G and MTHFR C677T gene polymorphisms and that the MTHFR 677T allele is associated with a reduced risk of hearing impairment, but only when the MTR A2756G genotype is a wild-type homozygote (AA).

We recently reported that the T allele of the MTHFR C677T gene polymorphism may be associated with an increased risk of SSNHL [29]. In the present analysis, the MTHFR 677 T allele decreased the risk of hearing impairment among the middle-aged and elderly, provided that the MTR A2756G genotype was AA. These apparently contradictory effects of the MTHFR C677T polymorphism on hearing are consistent with the results of Durga et al [30]. They examined the association between elevated plasma homocysteine level and its determinants with hearing levels in middle-aged and elderly subjects. Their hypothesis was that an elevated concentration of homocysteine and its determinants, including the MTHFR 677TT genotype, would be associated with poor hearing. Contrary to their hypothesis, the results demonstrated that MTHFR 677TT homozygotes had 5 dB (*p *= 0.037) and 2.6 dB (*p *= 0.021) lower PTA-high and PTA-low hearing thresholds, respectively, than subjects with a 677C allele when their folate status was above the median. They found that high serum folate concentrations were associated with better hearing in MTHFR 677TT homozygotes, whereas high serum folate concentrations were associated with poorer hearing in CC homozygotes. In the present study, the MTHFR variant allele conferred an advantage with respect to hearing when the MTR A2756G genotype was the wild type, which probably maintains MTR enzymatic activity at a higher level than in the variant [24]. Studies of carcinogenesis showed that an impaired MTR reaction, as observed in vitamin B_12 _deficiency or in cases of chronic ethanol ingestion, results in the continuous conversion of 5,10-methylene-THF to 5-methyl-THF. Thus, a substantial proportion of cellular folate is converted into a metabolically unavailable form, which results in a functional folate deficiency [34]. The MTR 2756A allele may prevent functional folate deficiency via MTR activity.

As referred to previously, the MTHFR 677T allele has favorable and unfavorable effects on human health. The conflicting effects of the MTHFR C677T polymorphism on carcinogenesis may be resolved as follows. When the dietary supply of folate and related nutrients is sufficient, individuals carrying variant MTHFR genotypes may be at reduced risk of cancer because high intracellular levels of 5,10-methyleneTHF may prevent imbalances in the nucleotide pool during DNA synthesis, thereby ensuring that DNA replication occurs with high fidelity [9,13]. DNA methylation is crucial for epigenetic modification of the genome, which is involved in the regulation of many cellular processes. Some MTHFR variants are associated with DNA hypomethylation [35]. The degree of DNA methylation modulates the risks of various cancers as a consequence of gene-environment interactions [36]. The MTHFR 677T allele may affect disease susceptibility in a beneficial or an adverse manner.

Durga et al. explained the etiology of AHRI in terms of mitochondrial damage [37]. It was proposed that damage to mitochondrial DNA accumulates over time and that once a critical threshold is attained, the mitochondria are rendered bioenergetically inefficient and cells undergo apoptosis. Individuals with the MTHFR 677TT genotype may have increased levels of thymidylate synthesis, which may directly reduce the probability of DNA mutations by ensuring a smaller pool of uracil and, hence, a decreased likelihood of uracil misincorporation into DNA. In addition, MTHFR 677TT homozygotes may have high levels of 10-formylTHF, which may protect mitochondrial integrity by reducing cytochrome c [38] and, because of its structural lability, may exert antioxidant-like effects [39].

This study has certain limitations. A major weakness of this study is that homocysteine and folate levels were available for only 22% of the cumulative data because of cost restriction. Also lab tests for vitamin B12 and/or methylmalonic acid were not performed, which are additional metabolic parameters potentially influenced by MTR enzymatic action. Current opinion is that the 677 C-to-T variant exerts its effect via an elevated homocysteine level as described as 'the homocysteine hypothesis' [14]. On the other hand, the homocysteine hypothesis and homocysteine-lowering therapeutic approaches to vascular disease have been questioned in recent years. Secondary prevention trials have failed to confirm the benefit of homocysteine-lowering therapy on cardiovascular disease [40,41]. Antoniades et al. proposed that folic acid and its circulating metabolite, 5-methylTHF, have direct effects on vascular function in humans, independent of their effects on plasma homocysteine level [42,43]. Therefore, data on changes in metabolites of the folate and homocysteine pathways and the status of B vitamins would have strengthened this study.

Another issue that has created some confusion is the seemingly contradictory effects of the MTHFR 677T allele on SSNHL vs. ARHI in the NILS-LSA study population. We reported that the MTHFR 677T allele may be associated with an increased risk of SSNHL and that it may be associated with a reduced risk of hearing impairment later in life if the individuals are wild-type homozygotes for MTR A2756G. No plausible explanation for this contradiction can be offered at this stage. However, given that one of the many hypotheses on the etiology of SSNHL involves impaired perfusion and ischemic damage to the cochlea, the MTHFR 677T allele may contribute to risk of an acute vascular event in the cochlea. On the other hand, the MTHFR 677T allele has certain advantages for the better-hearing ear in homozygous MTR 2756AA individuals, which are independent of plasma total homocysteine level. Bilateral hearing impairment as defined by the World Health Organization requires the presence of a hearing impairment in the better-hearing ear. Therefore, the better-hearing ear was used in our study in order not to overestimate individuals with only one affected ear as subjects with ARHI. The contribution of the MTHFR 677T allele to the affected ear in SSNHL may differ from its contribution to the better-hearing ear in middle-aged and elderly people.

Although genetic contributions to ARHI have been increasingly reported, significant associations between gene polymorphisms and a reduced risk of ARHI are rare. The results of this study may help to elucidate the complex mechanisms responsible for ARHI. This is an observational epidemiological design, which helps us to understand the factors that determine the presence or absence of disorder. Hypotheses derived from our results need to be verified by further basic and applied researches.

## Conclusions

The present study has found that the MTHFR 677T allele has a favorable effect on a risk of hearing impairment in the middle-aged and elderly population, only when the individuals were wild-type homozygotes for MTR A2756G. In addition, the favorable impact of the MTHFR 677T allele on the risk of developing hearing impairment in the MTR 2756AA genotype was independent of blood folate and homocysteine levels. These results may contribute to our understanding of the pathology of ARHI.

## Authors' contributions

YU carried out the statistical analyses and drafted the manuscript. HS and FA designed the study, collected the samples, and managed the overall project. YU, SS and TN implemented cooperation in conducting auditory section of the study.

All authors contributed to the writing of the manuscript, read and approved a final draft.

## Pre-publication history

The pre-publication history for this paper can be accessed here:

http://www.biomedcentral.com/1471-2350/12/35/prepub

## Supplementary Material

Additional file 1 **Supplemental figure S1. Simplified scheme of the role of MTR and MTHFR in folate metabolism and one-carbon transfer reactions**. The file contains a scheme which provides a comprehensible information regarding favorable and unfavorable effects of MTHFR 677T allele on biological activity. Click here for file
