# Supplementary material for: Hearing impairment risk and interaction of folate metabolism related gene polymorphisms in an aging study
Source: BMC Med Genet. 2011 Mar 7;12:35. doi: 10.1186/1471-2350-12-35 (PMC3063203; doi:10.1186/1471-2350-12-35)
Supplement: Additional file 1 — Supplemental figure S1. Simplified scheme of the role of MTR and MTHFR in folate metabolism and one-carbon transfer reactions. The file contains a scheme which provides a comprehensible information regarding favorable and unfavorable effects of MTHFR 677T allele on biological activity. [file 1471-2350-12-35-S1.PPTX]

## Slide 1
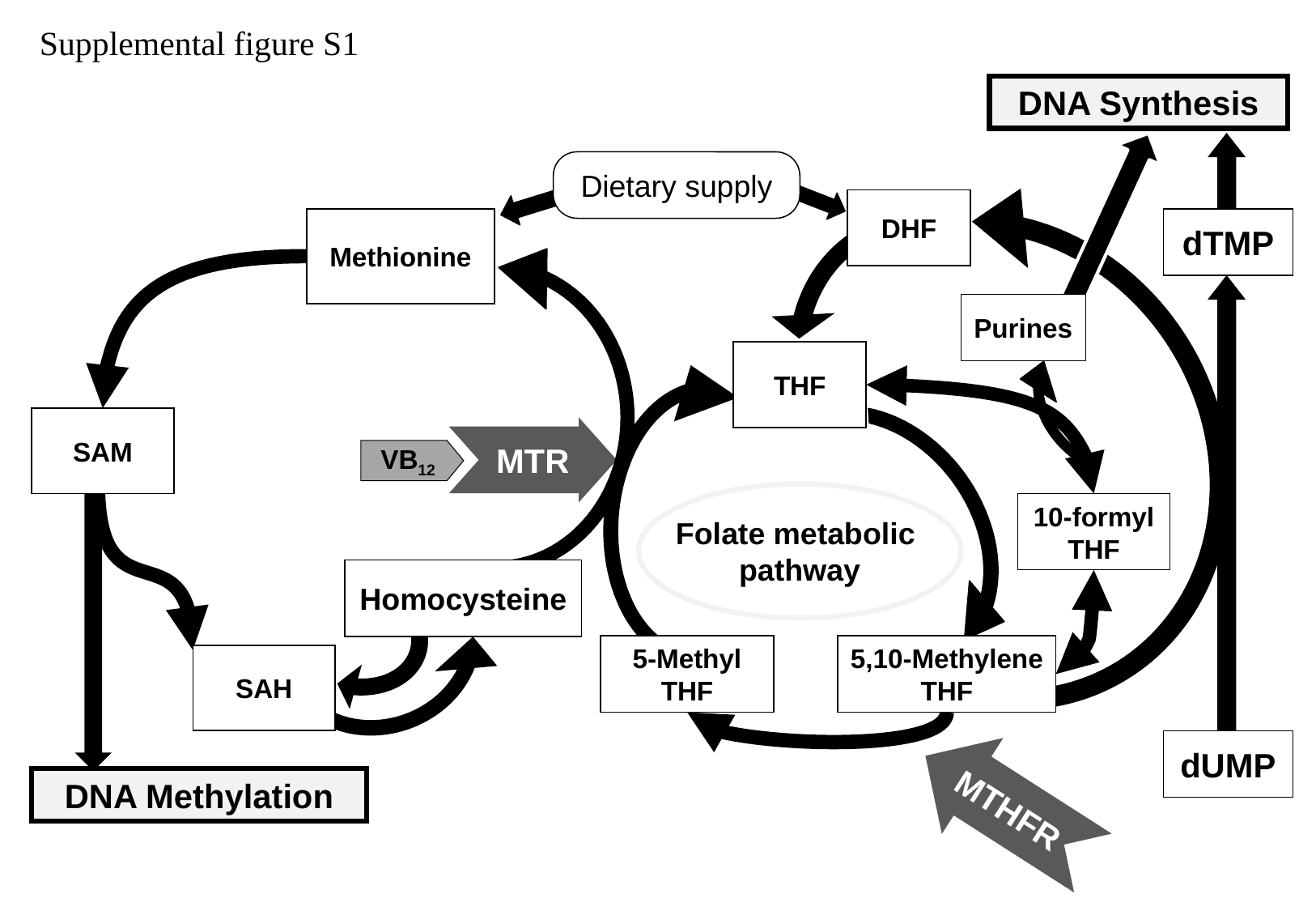

Supplemental figure S1
DNA Synthesis
Dietary supply
DHF
Methionine
dTMP
Purines
THF
SAM
MTR
VB12
Folate metabolic
pathway
10-formyl
THF
Homocysteine
5-Methyl
THF
5,10-Methylene
THF
SAH
dUMP
MTHFR
DNA Methylation
